# Peroral Endoscopic Removal of a Regurgitated Giant Polisegmented Fibrovascular Polyp of the Esophagus

**DOI:** 10.1155/DTE.7.197

**Published:** 2001

**Authors:** Robert Paczona, Laszlo Ivan, Jozsef Jori, Bela Ivanyi

**Affiliations:** 1 Department of Oto-Rhino-Laryngology Head and Neck Surgery Faculty of General Medicine University of Szeged, Tisza Lajos krt. 111 Szeged H-6725 Hungary; 2 Department of Pathology Faculty of General Medicine University of Szeged Tisza Lajos krt. 111 Szeged H-6725 Hungary

## Abstract

*Background*: Giant fibrovascular polyps (FVP) are relatively rare benign neoplasm of the upper esophagus and hypopharynx. Without previous history, their diagnosis might be difficult as the endoscopic findings are sometimes misinterpreted

*Materials and methods*: The present report describes a case, in which the patient regurgitated his giant polypoid mass into his mouth and captured it between his teeth and buccal surface until the emergency endoscopic removal

*Results*: After one-year of follow-up, the patient is going well, without recurrence of his polyp

*Conclusion*: Although the adequate therapy for these lesions is mainly the open surgical resection, most often via cervical esophagotomy, in our case the polyp was removed successfully by peroral endoscopic operation.


*Diagnostic and Therapeutic Endoscopy*, Vol. 7, pp. 197–201
Reprints available directly from the publisher
Photocopying permitted by license only

© 2001 OPA (Overseas Publishers Association) N.V.
Published by license under
the Harwood Academic Publishers imprint,
part of Gordon and Breach Publishing
a member of the Taylor & Francis Group.
All rights reserved.

### Peroral Endoscopic Removal of a Regurgitated Giant Polisegmented Fibrovascular Polyp of the Esophagus

ROBERT PACZONA[a], LASZLO IVAN[a,*], JOZSEF JORI[a] and BELA IVANYI[b]

[a]*Department of Oto-Rhino-Laryngology, Head and Neck Surgery, Faculty of General Medicine, University of Szeged, H-6725, Szeged, Tisza Lajos krt. 111, Hungary;* [b]*Department of Pathology, Faculty of General Medicine, University of Szeged, University of Szeged, H-6725, Szeged, Tisza Lajos krt. 111, Hungary*

*(Received 11 October 2001; Revised 10 December 2001; In final form 10 December 2001)*

*Background*: Giant fibrovascular polyps (FVP) are relatively rare benign neoplasm of the upper esophagus and hypopharynx. Without previous history, their diagnosis might be difficult as the endoscopic findings are sometimes misinterpreted.

*Materials and methods*: The present report describes a case, in which the patient regurgitated his giant polypoid mass into his mouth and captured it between his teeth and buccal surface until the emergency endoscopic removal.

*Results*: After one-year of follow-up, the patient is going well, without recurrence of his polyp.

*Conclusion*: Although the adequate therapy for these lesions is mainly the open surgical resection, most often via cervical esophagotomy, in our case the polyp was removed successfully by peroral endoscopic operation.

*Keywords*: Benign tumor; Endoscopy; Esophagus neoplasm; Fibrovascular polyp

### INTRODUCTION

Benign polypoid formation of the esophagus are relatively rare. This rare occurrence and the diversity of clinical symptoms, which they present, make their diagnosis sometimes very difficult. They can reach silently tremendous size without evoking any symptoms. Usually, the diagnosis can be established at time of their spectacular manifestations, such as regurgitation a fleshy mass into the mouth. Some cases were reported, when giant polyps caused airway obstruction, asphyxia, and sudden death due to the impactation into the larynx and upper respiratory tract. Although the appropriate treatment of such lesions is usually open surgical procedure, in the present report a case is described, in which this enormous size benign esophageal tumor was extirpated successfully by peroral endoscopic surgery.

*Corresponding author. Tel.: +36-62-545-310. Fax: +36-62-420-141.

### CASE REPORT

A 62-year-old man was transferred into the emergency unit of our university 1st Internal Medicine Department. Before the presentation of his disease, he had drunk moderate quantity of beer, he had an acute choking sensation, and suddenly he vomited the previously consumed alcohol mixed with gastric debris and fresh blood clot. At the end of this eructation, he delivered a fleshy mass into his oral cavity. He was not able to reswallow this mass, which was then captured between his left buccal mucosa and his teeth (Fig. 1).

Previously, he had symptoms of dysphagia, reflux, and substernal and cervical discomfort on swallowing. Three months earlier, he underwent an upper fibreoptic endoscopy in other hospital. Examination showed a deviated esophagus, with superficial mucosal inflammation, the findings were interpreted as a non-specific esophagitis. Anti reflux treatment was administered and as the patient's complaints were resolved within few weeks, no further esophageal examination was performed. In his past medical history, repeated attempts of electrocardioversion for atrial fibrillation were revealed. He was treated by beta-blockers and acetylsalicylic acid, his family history was uneventful.

The otolaryngology department was consulted, and the patient was transferred to our department. On fiberoptic laryngeal examination, behind the dominant intraoral mass, two minor bulky tumors were visualized. These masses were attached by a thin stalk, which extends along to the left lateral pharyngeal wall, disappearing into the esophageal inlet. Laryngeal examination revealed no abnormalities, true vocal cords was mobile, and no obstruction of the larynx was found.

He was taken to the operating room, where he underwent an endoscopic laryngeal and esophageal examination under general anesthesia. A Weerda-type bivalved adjustable laryngoscope (STORZ Company, Tuttlingen, Germany) tube was introduced into the hypopharyngeal region and the origin of the polyp was perfectly visualized. The polypoid mass was found to arise from a mucosal stalk that was attached in the midline to the cervical esophagus at the level of cricopharyngeal muscle. Consequence of a previous experience with a similar disease occurred 20 years earlier at our department [1], no further diagnostic imaging procedures was demanded, and a peroral endoscopic removal was decided to avoid the invasive cervical esophagotomy. First, to prevent uncontrolled bleeding, double suture ligation of the stalk, 1 cm below his site of origin of the upper esophagus, was

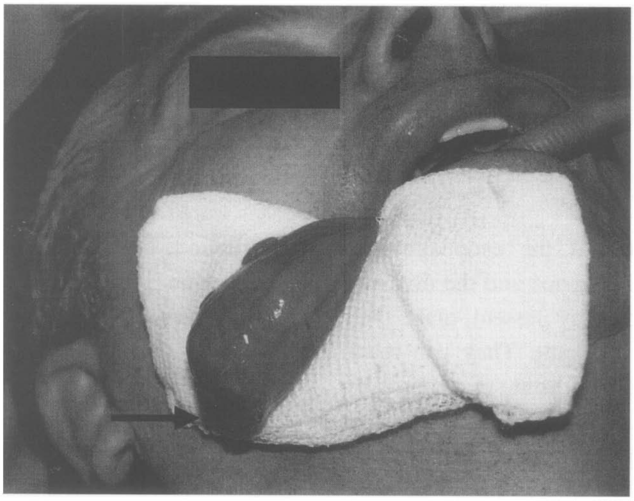

FIGURE 1 Clinical presentation of our case. Intra-operative delivery of the polyp from the mouth before the excision. Arrow: ulceration of the distal tip of the polyp.

performed under operating microscope. Secondly, the polyp was encircled with an oval snare, than elektrocoagulation was carried out to transect the stalk of the polypoid mass.

### PATHOLOGIC FINDINGS

Grossly, the mass had a pink-tan appearance. The lesion was composed of one large distal polyp measured $12.0 \times 6.0 \times 3.0\,cm^3$ attached with his feeding stalk to two proximal segmented polypoid mass measured $10.0 \times 4.0 \times 2.0\,cm^3$. The whole giant polyp length measured 22 cm. Light microscopically, a benign tissue proliferation was observed. The surface was covered by thickened and partly ulcerated squamous epithelium. The lesion was composed of blood vessels, fibrous tissue, and accentuated mainly beneath the surface, inflammatory infiltrates. The vascular component contained arteries, large number of prominent open capillaries lined by a single layer of plump endothelial cells, and small veins. The large vessels had an incomplete and irregular smooth muscle coat, and not rarely, they lacked elastic fibers. The stroma varied from loose and oedematous, with fibroblasts and numerous mast cells, to a dense acellular and collagenized tissue. The infiltrates contained mainly lymphocytes, admixed with plasma cells, and mononuclears (Fig. 2). The lesion was interpreted as inflammatory fibrovascular polyp.

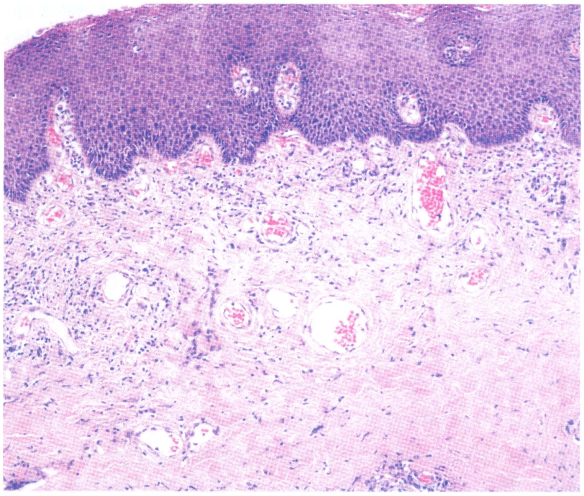

FIGURE 2 Microscopic picture showing fibrovascular stroma, focal flat and several muscular vessels (light microscopic picture, HE staining, 10 × magnification).

### DISCUSSION

Benign esophageal tumors are relatively rare, they can be subdivided into three subgroups; as intramural, extramural and intraluminal lesions. Leiomyomas are the most common tumors of the intramural group, frequently asymptomatics. The extramural tumors cause typically compression of the esophagus itself and the adjacent organs such a trachea, with the main symptoms of dysphagia and dyspnea. The intraluminal tumor's group includes papillomas and polyps [2,3].

In the terminology of intraluminal polyps several confused and various terms exist, such as fibroma, fibrolipoma or fibromixoma of the esophagus. Histologically, all entities have the same appearance; they contain fibrous tissue with vascular component and variable amounts of myxoid and adipose tissues. Currently, the term "fibrovascular polyp" should be applied, since the above mentioned variations do not seemed to be distinct entities and show the same clinical symptoms [3,4].

The fibrovascular polyps (FVP) develop characteristically in the upper third of the esophagus, in the retrocricoid region, inferior to the crico-pharyngeal muscle. A common sites of origin of these tumors the area of the Laimer's triangle, where the muscular support of the esophageal wall is relatively deficient. Polyps begin as the submucosal thickenings, become later nodular changes and may be elongated by the peristaltic and traction forces of the esophagus during the swallowing process. The peristaltic forces can form the polyps into giant proportions [3].

Correct diagnosis of FVP are very difficult as most of the patients remain asymptomatics until the polyps reach significant, sometimes enormous dimensions. Symptoms are less characteristics, the most frequent complaint is dysphagia, while cervical, substernal discomfort, globus sensation are highly related to esophageal obstruction syndrome. Bleeding from the ulceration of the distal tip of the FVP resulted by caustic burns from refluxed gastric contents has also been described [5].

Regurgitation of the polyps into the pharynx and the oral cavity is frequent, the half of cases manifested by this spectacular manner [4–7]. This kind of exteriorization of the FVP may be a life-threatening situation, due to a potential cause of sudden laryngeal obstruction, suffocation, and asphyxia. Several cases have been reported, when the regurgitated polyp caused respiratory problems, and some of them could be only resolved by temporary tracheotomy [3]. In our case, the patient captured this regurgitated fleshy mass between his buccal mucosa and teeth. As the stalk of this poly-segmented polyp was relatively thin, and the lesion was too bulky to fall into the larynx, no respiratory distress was observed during the eructation.

Radiographic imaging, as the modified barium swallow images is useful in the evaluation of esophageal polyps. Intraluminal filling defect with regular borders is characteristic of a pedunculated polypoid mass. CT and MRI studies are more helpful to precise these lesions [8].

Endoscopic evaluation of esophageal lesions is mandatory before the definitive treatment. The squamous epithelium overlying the polyp is identical in appearance and often indistinguishable from the mucosa of normal esophagus. The mobility of the lesion may also error the interpretation of the disease. Overlooking and misdiagnosing an intraluminal polyp with endoscopy occurred very frequently [3], as it happened in our case also. Prior to the spectacular manifestation of his polyp, our patient underwent a flexible upper gastrointestinal endoscopy.

The definitive treatment for these polyps is surgical excision. External lateral pharyngotomy allows for better visualization of the origin of polyp's stalk, and the suture ligation and excision of the lesion [2,4–6,9,10]. For those polyps, whose origin of their stalk situated lower than the crico-pharyngeus muscle, transthoracic esophagotomy is required [11]. Endoscopic peroral removal has been considered hazardous and dangerous method of polyp ablation with concerns regarding bleeding [12]. Some authors emphasized, that endoscopic removal is only possible in cases of small polyps, while more larger lesions are advocated to toward external surgical approach [1,4,12]. In our case, the most reasonable therapeutic strategy was elective endoscopic polypectomy on the basis of precise diagnosis. As the polyp is exteriorized and captured outside, and as we have been already operated a patient earlier with a good result, the approach was obvious, endoscopic oriented peroral resection. The visualization of the operating field with the bivalved adjustable laryngoscope tube was no more limited compared with open surgical procedure and proved exceptionally useful to explore the origin of the stalk in the retrocricoid region. It provided enough space to prevent uncontrolled bleeding, using suture ligation of the stalk before the snare cautery excision.

After one-year of follow-up, our patient is going well, without recurrence of his polyp.

### References

[1] Kaucsek, H. and Csepregi, E. (1980) "Diagnosztikus nehezseget okozo nyeles polyp a nyelocsoben. [Pedunculated polyp in the esophagus-causing diagnostic difficulty]", *Ful-orr-gegegyogyaszat* **26**, 46–49.

[2] Avezzano, E.A., Fleischer, D.E., Merida, M.A. and Anderson, D.L. (1990) "Giant fibrovascular polyps of the esophagus", *Am. J. Gastroenterol.* **85**(3), 299–302.

[3] Owens, J.J., Donovan, D.T., Alford, E.L., *et al.*, (1994) "Life-threatening presentations of fibrovascular esophageal and hypopharyngeal polyps", *Ann. Otol. Rhinol. Laryngol.* **103**, 838–842.

[4] Siddins, M. and Cade, R. (1991) "Fibrovascular polyp of the esophagus", *Aust. NZ. J. Surg.* **61**, 237–240.

[5] Vrabec, D.P. and Colley, A.T. (1983) "Giant intraluminal polyps of the esophagus", *Ann. Otol. Rhinol. Laryngol.* **92**, 344–348.

[6] Patel, J., Kieffer, R.W., Martin, M. and Avant, G.R. (1984) "Giant fibrovascular polyp of the esophagus", *Gastroenterology* **87**, 953–956.

[7] Wu, M.H., Chuang, C.M. and Tseng, Y.L. (1998) "Giant intraluminal fibrovascular polyp of the esophagus", *Hepato-Gastroenterology* **45**, 2115–2116.

[8] Levine, M.S., Buck, J.L., Pantograg-Brown, L., Buetow, P.C., Hallman, J.R. and Sobin, L.H. (1996) "Fibrovascular polyps of the esophagus: clinical, radiographic, and pathologic findings in 16 patients", *AJR* **166**, 781–787.

[9] Eliashar, R., Saah, D., Sichel, J.Y. and Elidan, J. (1998) "Fibrovascular polyp of the esophagus", *Otolaryngol. Head Neck Surg.* **118**(5), 735–736.

[10] Belafsky, P., Amedee, R. and Zimmerman, J. (1999) "Giant fibrovascular polyp of the esophagus", *South. Med. J.* **92**, 428–431.

[11] Boggi, U., Viacava, P., Naccarto, A.G., *et al.*, (1997) "Giant pedunculated liposarcomas of the esophagus: literature review and case report", *Hepato-Gastroenterology* **44**, 398–407.

[12] Burdick, J.S., Seidel, R. and Lindberg, G. (1999) "Endoscopic removal of an esophageal fibrovascular polyp", *Endoscopy* **31**(5), 401–404.

